# Hydroxybenzoic acid isomers and the cardiovascular system

**DOI:** 10.1186/1475-2891-13-63

**Published:** 2014-06-19

**Authors:** Bernhard HJ Juurlink, Haya J Azouz, Alaa MZ Aldalati, Basmah MH AlTinawi, Paul Ganguly

**Affiliations:** 1 Department of Anatomy, College of Medicine, Alfaisal University, Riyadh, Kingdom of Saudi Arabia; 2 Department of Anatomy & Cell Biology, University of Saskatchewan, Saskatoon, SK, Canada; 3 College of Medicine, Alfaisal University and Adjunct Scientist, King Faisal Specialized Hospital and Research Centre, Riyadh, Kingdom of Saudi Arabia

**Keywords:** Antioxidant enzymes, Atherosclerosis, Dyslipidemia, Hydroxycarboxylic acid receptors, Hypertension, Inflammation, Lipolysis, Nrf2, Phytochemicals, Oxidative stress, Dihydroxybenzoic acid, Cardiovascular diseases, Food products, Pharmacologically-active compounds

## Abstract

Today we are beginning to understand how phytochemicals can influence metabolism, cellular signaling and gene expression. The hydroxybenzoic acids are related to salicylic acid and salicin, the first compounds isolated that have a pharmacological activity. In this review we examine how a number of hydroxyphenolics have the potential to ameliorate cardiovascular problems related to aging such as hypertension, atherosclerosis and dyslipidemia. The compounds focused upon include 2,3-dihydroxybenzoic acid (Pyrocatechuic acid), 2,5-dihydroxybenzoic acid (Gentisic acid), 3,4-dihydroxybenzoic acid (Protocatechuic acid), 3,5-dihydroxybenzoic acid (α-Resorcylic acid) and 3-monohydroxybenzoic acid. The latter two compounds activate the hydroxycarboxylic acid receptors with a consequence there is a reduction in adipocyte lipolysis with potential improvements of blood lipid profiles. Several of the other compounds can activate the Nrf2 signaling pathway that increases the expression of antioxidant enzymes, thereby decreasing oxidative stress and associated problems such as endothelial dysfunction that leads to hypertension as well as decreasing generalized inflammation that can lead to problems such as atherosclerosis. It has been known for many years that increased consumption of fruits and vegetables promotes health. We are beginning to understand how specific phytochemicals are responsible for such therapeutic effects. Hippocrates’ dictum of ‘Let food be your medicine and medicine your food’ can now be experimentally tested and the results of such experiments will enhance the ability of nutritionists to devise specific health-promoting diets.

## Introduction

The identification of salicin and salicylic acid as the chemical compounds that gave willow bark its analgesic and antipyretic properties initiated the development of the modern pharmaceutical industry and pharmaceuticals now dominate the therapeutic interventions of modern Western (allopathic) medicine. During the past few centuries there have been major breakthroughs in understanding the role of foods in the maintenance of life, including: i) the identification of carbohydrates, lipids and proteins and their roles in maintaining the metabolic machinery of our bodies, ii) the identification of vitamins and minerals and their roles in metabolism. The past century also led to major breakthroughs in understanding cellular signaling pathways and the control of gene expression. We are now beginning to understand how components in our foods, mainly certain phytochemicals, are affecting cellular signaling thereby influencing metabolism as well as gene expression. We are, thus, on the cusp of the third era of nutrition where we will understand the roles that particular phytochemicals can play in altering metabolism and gene expression that leads to better health [1]. In this review we consider the possible therapeutic effects of hydroxybenzoic acids that chemically are closely related to the first identified pharmaceuticals, salicin and salicylic acid. These compounds either decrease oxidative stress and inflammation through promotion of the expression of antioxidant enzymes or they inhibit adipocyte lipolysis through activation of hydroxycarboxylic acid receptors, thereby potentially promoting better plasma lipid profiles. Of course, everything is double-edged and phytochemicals may also affect the activity and/or expression of the phase 1 enzymes that metabolize xenobiotics, including drugs. The past has shown us that if one were taking the calcium channel blocker felodipine it becomes important for one’s health not to consume grapefruit [2]. Thus, if one were to alter diet to increase intake of particular phytochemicals, it becomes important to know how such phytochemicals affect the function of phase 1 enzymes.

A major aim of this review is to interest researchers in the area of nutrition to investigate how phytochemicals influence cellular signaling and gene expression so that rapid progress can be made in the science of Hippocrates’ dictum: ‘let food be your medicine and medicine your food’.

## Review

### Discovery of salicin and salicylic acid

**Figure 1:**
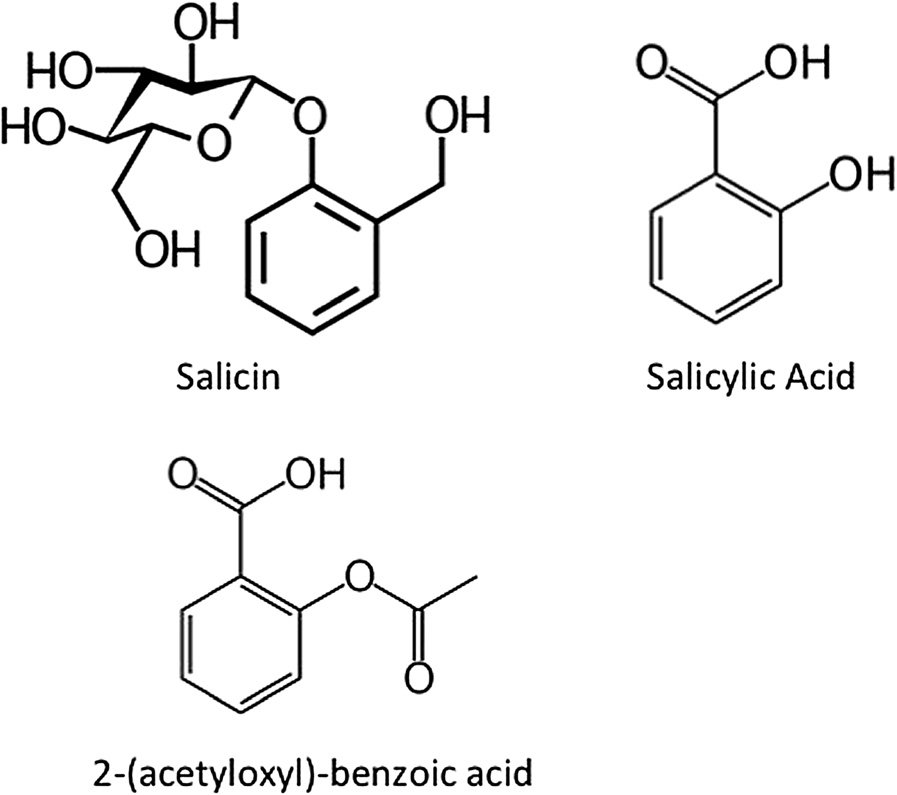
**Structures of salicin, salicylic acid and acetylsalicylic acid (2-[acetyloxyl]-benzoic acid).** Chemical diagrams taken from Wikimedia Commons.

The first pharmacologically-active drugs isolated from a herbal preparation were identified during the nineteenth century [3]. These were the analgesics salicin (2-(hydroxymethyl)phenyl-β-D-glucopyranoside) and its metabolite salicylic acid (2-hydroxybenzoic acid) (Figure 1): these were obtained from willow bark extracts [3]. The analgesic and antipyretic activities of willow bark extracts were known far earlier, being mentioned in Egyptian and Sumerian texts [4]. During the latter part of the nineteenth century salicylic acid was acetylated to form the more gastrointestinal-friendly non-steroidal anti-inflammatory drug acetylsalicylic acid (ASA or 2-[acetyloxyl]benzoic acid), commonly referred to as aspirin. Although used since the end of the nineteenth century the mechanisms of action of aspirin were only beginning to be discovered in the 1970s where it was demonstrated that aspirin inhibited the action of cyclooxygenase (COX) thereby inhibiting the synthesis of pro-inflammatory eicosanoids [5]. More recently it has been shown that aspirin also promotes the acetylation of COX2 resulting in the promotion of the synthesis of 15-hydroxyeicosatetraenoic acid that is converted into the anti-inflammatory eicosanoid 15-epi-lipoxin A4 [6].

Following the discovery of salicin and synthesis of aspirin a large pharmaceutical industry arose around the identification and isolation of the pharmacologically-active compounds present in herbal medicines, with often modification of such active compounds to form the drugs currently in clinical use. Soon pharmaceuticals dominated the therapeutic interventions of Western (allopathic) medicine. Forgotten was Hippocrates’ dictum: “Let food be your medicine and medicine your food”. This article is primarily aimed at discussing the possible roles of the isomers of dihydroxybenzoic acid, that are present in certain fruits and vegetables, in preventing cardiovascular diseases.

### Pharmacologically active compounds in foods that we eat

Recently it has become recognized that pharmacologically active compounds are present not only in herbal products but also in many of our foods; hence, foods, in principle, could, as stated by Hippocrates, be used in preventing, if not treating, many diseases, particularly diseases related to lifestyle that become more common with age.

One of the earliest identified pharmacologically active components in food is the isothiocyanate sulforaphane, which is a metabolite of the sulforaphane glucosinolate, also known as glucoraphanin [7]. Sulforaphane glucosinolate is present in crucifers and is present in very high levels in broccoli sprouts of particular cultivars [8]. Sulforaphane is a very potent activator of nuclear factor (erythroid-derived-2)-like-2 [Nrf2]) [9] through oxidation of the thiols of the protein Kelch-ECH-Associated Protein 1 (Keap1) that normally sequesters Nrf2 in the cytoplasm [10]. Nrf2 promotes the expression of genes whose protein products either promote scavenging of oxidants or decrease the likelihood of oxidant production [11,12]. A more oxidizing environment results in many physiological problems. For example, a more oxidizing environment results in readier activation of the transcription factor complex nuclear factor kappa B (NFκB) [13] that, in turn, promotes expression of pro-inflammatory genes.

Broccoli sprouts rich in sulforaphane glucosinolate have been shown to reduce oxidative stress and inflammation in hypertensive rats thereby promoting better endothelial function and lower blood pressure [14]. A similar effect is seen when rats are given sulforaphane by gavage [15], indicating that the health-promoting effects of broccoli sprouts is due to the sulforaphane metabolite of the glucosinolate rather than some other component that may be present. Furthermore, the less oxidative stress and inflammation in pregnant hypertensive rats fed with broccoli sprouts results in less oxidative stress, inflammation and elevated blood pressure in the offspring even when the offspring do not have a diet rich in Nrf2 activators [16]. Thus, diet can have positive effect on fetal determinants of adult health. We, clearly, are now in the third era of nutritional research and are beginning to understand how specific phytochemicals affect cell signaling and gene expression and, thereby, health [1].

A concern may arise whether the increase in consumption of foods that have increased Nrf2 activity may be harmful. As far as sulforaphane glucosinolate is concerned phase 1 clinical trials in human have indicated no ontoward effect on liver and thyroid function when ingesting broccoli sprout extracts rich in sulforaphane glucosinolate [17]. Furthermore, another human trial in type 2 diabetics has shown that consuming a broccoli sprout extract containing either 112.5 or 225 micromoles of sulforaphane glucosinolate significantly decreased fasting glucose and insulin levels [18], demonstrating that food sources can be used as a medicine. Finally, the Juurlink laboratory has shown that intake of 10 μmol sulforaphane/kg body weight by gavage for 4 months had no detectable negative effect on the Stroke-prone spontaneously hypertensive rats [15], nor did consumption of broccoli sprouts containing 5–10 μmol sulforaphane equivalents/Kg body weight have any effect on the normal physiology Sprague Dawley rats [14]; thus, compounds that are Nrf2 activators appear to have physiological effects only in individuals that are under oxidative stress. Intake of sulforaphane tips the cell to a more normal redox state thereby decreasing the probability of problems related to inflammation. For a more detailed look at how the Nrf2 system influences cardiovascular health, please see [12].

Surprisingly, although there are over a thousand papers examining the positive effects of sulforaphane in preventing cancer, treating cancer, decreasing oxidative stress or treating conditions with an underlying oxidative stress and inflammatory component, there are no toxicology studies reported for this compound. Sulforaphane is an electrophile and like other electrophiles it oxidizes thiols. However, unlike other electrophiles such as dimethyl fumarate, sulforaphane - as well as certain other phytochemicals - has the particular electro-geometry that allows oxidation of Keap1 thiols at submicromolar concentrations. Thus, 50 nM sulforaphane has the same ability to increase Nrf2-inducible protein expression [19] as 25 μM dimethyl fumarate [20]: in other words, 500 times as many thiols are oxidized by dimethyl fumarate, a drug recently approved as a treatment for multiple sclerosis [21], to obtain the same Nrf2 activation as 50 nM sulforaphane.

Keap1 thiols are not the only thiols oxidized by sulforaphane and one might anticipate that sulforaphane ought to interfere with many cellular functions. In an attempt to address this, Piberger and colleagues examined the ability of sulforaphane to release zinc from a synthetic peptide that resembled the zinc-binding domain of xeroderma pigmentosum A [22]. They demonstrated that sulforaphane at concentrations of 50 μM or greater caused zinc release from the peptide; however, they also demonstrated that lower levels of sulforaphane (5 μM) interfered with the xeroderma pigmentosum A-dependent nucleotide excision repair. It is unlikely that plasma levels of sulforaphane can reach 5 μM through dietary intake of sulforaphane glucosinolate. Indeed, male spontaneously hypertensive stroke-prone rats fed daily a dried broccoli sprouts containing 14.5 micromoles of sulforaphane equivalent only achieved a plasma level of 0.5 μM dithiocarbamate [23], the sulforaphane metabolite. One must also keep in mind that unlike in cell culture studies where there is a constant concentration of the compound of interest, dietary intake of sulforaphane, whether through food consumption or through gavage, results in fluctuating plasma levels where peak concentrations can result in sustained elevations of anti-oxidant proteins through activation of the Nrf2 system but only transient inactivation of the function of proteins such as xeroderma pigmentosum A. Clearly, there is an abundance of evidence, both epidemiological and experimental that is in support of the ability of sulforaphane’s health-promoting activities [24].

### Concerns with increasing consumption of pharmacologically active compounds found in our foods

The knowledge of which particular cultivar one is consuming can be important. For example, various cultivars of broccoli and other crucifers have different glucosinolate profiles and a major concern with glucosinolates is that some of them are goitrogenic [25]; hence, it is important to ensure that one is increasing sulforaphane glucosinolate consumption that one does not consume significant quantities of goitrogenic glucosinolates. In the studies by the Juurlink laboratory the Calabrese variety of broccoli was used since this cultivar has high levels of sulforaphane glucosinolate and other Nrf2-activating glucosinolates but low levels of the goitrogenic glucosinolates [14].

Phytochemical compounds may have more than one mechanism of action. Another major concern is effects of phytochemicals on the expression and/or activity of the drug metabolizing phase 1 enzymes, for example, the cytochrome P450s (CYPs). Altering phase 1 enzyme activity can affect drug metabolism. For example, the flavanone naringenn activates Nrf2 [26] but it is also a competitive inhibitor of CYP3A4 [27]. CYP3A4 is involved in the metabolism of many commonly used drugs. For example, CYP3A4 is involved in the metabolism of felodipine, a calcium channel blocker [28]. If one is taking felodpine, consuming increased amounts of naringenin and furanocoumarins present in grapefruit juice may cause dangerous elevations in the plasma level of felodipine resulting in dangerously low blood pressure. Other Nrf2 activators, such as sulforaphane, also have effects on phase 1 enzyme expression or activity [29], for example, sulforaphane inhibits CYP3A4 gene expression and inhibits CYP1A2 and CYP2E1 [30]. Clearly, if one is on a medication it becomes important to know the effects of consuming increasing amounts of foods with pharmacologically active components. Physicians are already aware that when administering the vitamin K epoxide reductase inhibitor warfarin, that the dosage required for the desired pharmacological effect is dependent upon dietary intake of green leafy vegetables that are rich in vitamin K [31]. Thus, altering diet to increase intake of phytochemicals that are pharmacologically active will make the life of a physician or nutritionist more complicated.

### The hydroxybenzoic acids

**Figure 2:**
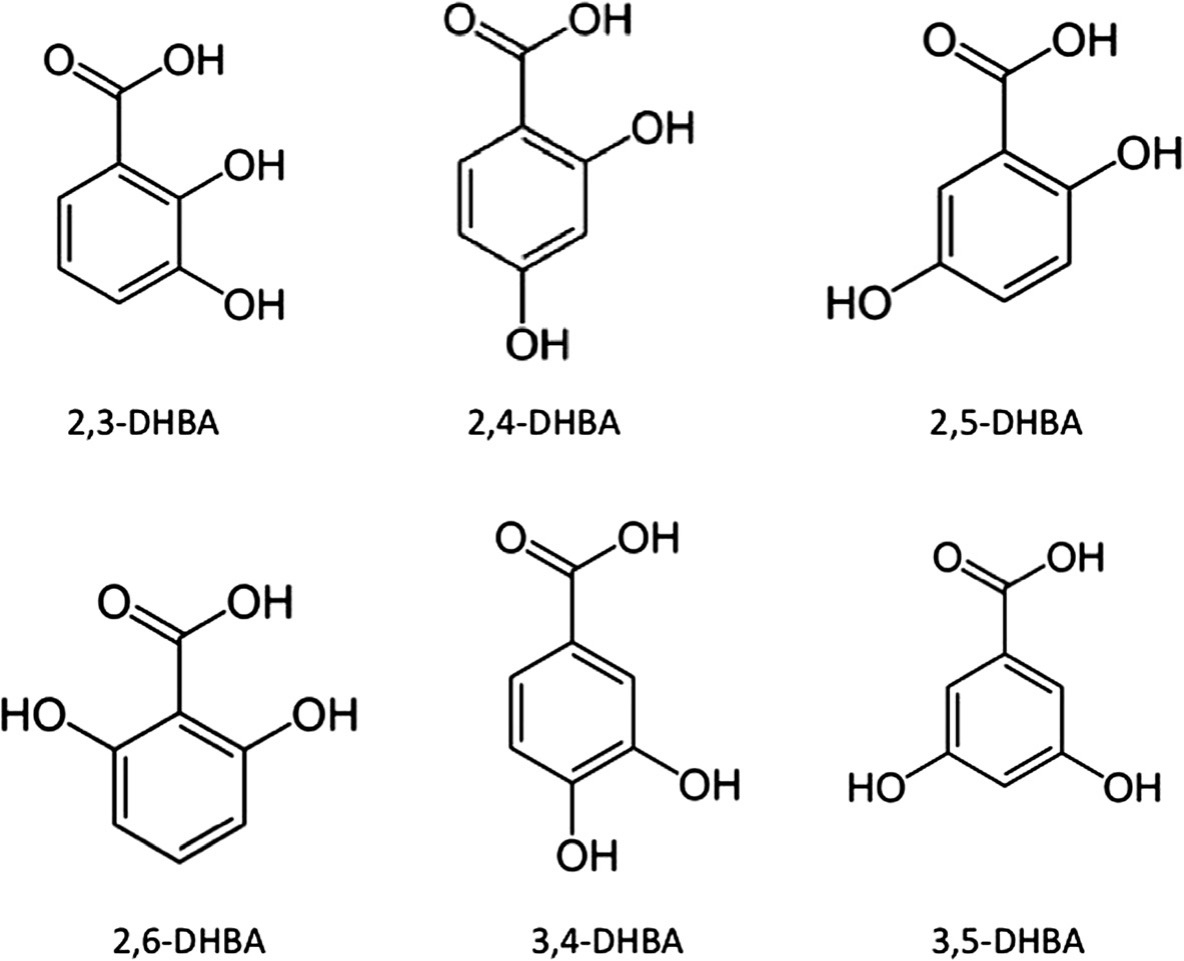
**Structures of the isomers of dihydroxybenzoic acid.** Chemical diagrams taken from Wikimedia Commons.

It is 185 years since Henri Leroux first isolated a pure crystalline form of salicin [3]. It seems timely to revisit this family of hydroxyphenols in the context of human health. There are a number of dihydroxybenzoic acid (DHBA) compounds, related to salicylic acid, that are also pharmacologically active, some of which are metabolites of salicylic acid. Their chemical formulae are outlined in [32] and given in Figure 2. The compounds include 2,3-DHBA (Pyrocatechuic acid or Hypogallic acid), 2,5-DHBA (Gentisic acid), 2,4-DHBA (β-Resorcylic acid), 2,6-DHBA (γ-Resorcylic acid), 3,4-DBHA (Protocatechuic acid) and 3,5-DHBA (α-Resorcylic acid) [32,33]. The hydroxybenzoic acids are phytochemicals that can be found in certain of foods and can be also be formed from polyphenols such as flavonoids by gut bacteria, e.g., [34]. Because they are hydroxylated phenolic compounds they all can scavenge oxidants such as free radicals via their hydroxyl groups [35], but this is not an important mechanism of action since essentially one hydroxylated phenolic compound can scavenge only one or two strong oxidants. Their more interesting properties are associated with their ability to modify cellular signaling processes that introduces a multiplier effect, one example is activation of the Nrf2 pathway that results in enhancement of multiple endogenous anti-oxidant mechanisms. We will focus on a few of these hydroxyphenolic compounds in this review.

#### ***2,3-DHBA (Pyrocatechuic acid)***

Pyrocatechuic acid is a metabolite of aspirin [33]. It is normally present in plasma even when there has been no intake of aspirin [36], indicating a dietary source of either 2,3-DHBA or a precursor molecule. 2,3-DHBA is present in several medicinal herbs, including, Madagascar rosy periwinkle [37] and *Boreava orientalis*[38] as well as in a number of fruits such as batoko plum commonly made into preserves in South and South-East Asia [39], avocados [40] and cranberries [41,42]. A major dietary source of pyrocatechuic acid is *Aspergillus-*fermented soy products, popular in Japan, that can contain more than 2 mmol 2,3-DHBA/L soy product [43].

2,3-DHBA decreases hydrogen peroxide-induced activation of the transcription factor complex nuclear factor kappa B (NFκB) that plays an important role in inflammation [44]. The mechanism of action for this effect may be simple scavenging of hydrogen peroxide [35] or possibly the activation of the antioxidant response; however, this latter mechanism of action has not been examined in this hydroxybenzoic acid metabolite, although other dihydroxybenzoic acids have this ability. Nor has there been examination of the possible effects of 2,3-DHBA on phase 1 enzyme expression and activity. Administration of 2,3-DHBA to a rat model of sepsis has been reported to decrease mortality when used in conjunction with gentamycin [45], likely through decreased tissue damage related to oxidative stress and associated inflammation. Relatively little is known about the distribution of 2,3-DHBA in the plant kingdom nor its mechanism of therapeutic action but both seem worthy of further investigation.

#### ***2,5-DHBA (gentisic acid)***

Gentisic acid like aspirin inhibits prostaglandin formation in response to lipopolysaccharides [46], presumably via inhibiting COX activity. This suggests that foods rich in gentisic acid may help decrease the probability of heart attacks due to clot formation. Gentisic acid also inhibits the oxidation of low-density lipoprotein and inhibits the formation of lipid hydroperoxides [47,48] and, thus, decreases the probability of atherogenesis. These effects of gentisic acid are usually attributed to its ability to scavenge free radicals and other oxidants; however, gentisic acid is also an Nrf2 activator [49] and this is the most likely relevant mechanism. In the study by Yeh and Yen [49], gentisic acid was introduced into the diet whereby rats consumed very high amounts of gentisic acid (650 μmol/Kg/day) - what plasma levels were achieved was not measured. Clearly, dose–response studies are required to determine whether gentisic acid activates the Nrf2 system at much lower dietary intakes. Also, at high concentrations gentisic acid is an aldose reductase inhibitor but the IC50 is over 200 μM [50], concentrations that are likely not attainable via the diet. How, and whether, gentisic acid affects phase 1 enzyme gene expression and activity is not known. CYP2E1 and CYP3A4 are involved in the metabolism of gentisic acid [51].

Gentisic acid is widely present in foods we consume, including cereals such as wheat and rye [52], actinidia (e.g., kiwi) fruit [53], aloe vera [54], a number of mushrooms [55] as well as other sources. For quantitative data on gentisic acid distribution in food sources see Table 1.

**Table 1:** Dietary sources of gentisic acid

**Source**	Content*	**References**
Apple	2 μmol/Kg	[56]
Beer	2 μmol/L	[57]
Bilberries	150 μmol/kg	[58]
Bitter melon, ripe	4,220 μmol/Kg	[59]
Blackberries	135 μmol/Kg	[58]
Blueberries, *V. arctostaphylos*	1.3 μmol/Kg	[60]
Currants, black	155 μmol/Kg	[58]
	75 μmol/kg	[56]
Gooseberries	42 μmol/kg	[56]
Grapes, white	2.9 μmol/kg	[56]
Honeysuckle, blueberried	150 μmol/Kg	[58]
Juneberries, European	220 μmol/Kg	[58]
Kiwi, common varieties	585 Mol/Kg	[53]
Kiwi, *A. Kolomikta*, ‘Dr Szymanowski’	27,610 μmol/Kg	[53]
Mulberries, black	112 μmol/Kg	[58]
Pear	2.5 μmol/Kg	[56]
Strawberries	118 μmol/Kg	[56]
Wine, red	3 μmol/L	[61]
Wine, white	1 μmol/L	[61]

*Values given are for the free phenolic as well as the phenolic derived from either esters or glycosides. All published values converted to μmoles per unit volume or per Kg fresh fruit (based upon an 85% water content).

#### ***3,4-DHBA (protocatechuic acid)***

Protocatechuic acid is widely distributed, in our foods being found in buckwheat [62], mustard [63], nipa palm nut [64], kiwi fruit [65], currents [66], blackberries and strawberries [67], Jujube fruit [68], chokeberries [69], mango [70]. In addition, it is also found in chicory, olives, dates, grapes, cauliflower, lentils, etc. [71]. For quantitative data on protocatechuic acid in food sources see Table 2.

**Table 2:** Dietary sources of protocatechuic acid

**Source**	Content*	**References**
Açai pulp	11.7 μmol/L	[72]
Apple	31 μmol/Kg	[56]
Avocado	2.4 μmol/Kg	[73]
Beer	3 μmol/L	[57]
Bilberries	111 μmol/Kg	[58]
Bitter melon, ripe	970 μmol/Kg	[59]
Blackberries	127 μmol/Kg	[58]
Blueberries, *V. arctostaphylos*	9.5 μmol/kg	[60]
Buckwheat, whole grain	600 μmol/Kg	[62]
Cauliflower, raw	29 μmol/Kg	Phenol-Explorer^†^
Chicory	1,090-1,415 μmol/Kg	Phenol-Explorer
Currants, black	78 μmol/Kg	[58]
	357 μmol/Kg	[56]
Dates, dried	320 μmol/kg	Phenol-Explorer
Eggplant, purple, raw	48 μmol/Kg	Phenol-Explorer
Garlic	23 μmol/Kg	[74]
Gooseberry	405 μmol/Kg	[56]
Grapes, white	22 μmol/Kg	[56]
Honeysuckle, blueberried	140 μmol/Kg	[58]
Juneberries, European	159 μmol/Kg	[58]
Kiwi juice	39 μmol/L	[65]
Lentils, dried, dehulled	4.5 μmol/Kg	Phenol-Explorer
Lentils, dried with hulls	9 μmol/Kg	Phenol-Explorer
Lingonberries	195 μmol/Kg	[75]
Mango pulp	2.5 μmol/Kg	[73]
Mangosteen pulp	91 μmol/Kg	[76]
Medlar, ripe	6 μmol/Kg	[77]
Mulberries, black	119 μmol/Kg	[58]
Oil, Açai – phenol rich	4 μmol/mL	[72]
Oil, olive – virgin	3-11.5 μmol/Kg	[78]
Olive, black, raw	390 μmol/kg	Phenol-Explorer
Olive, green, raw	43 μmol/Kg	Phenol-Explorer
Onion, red	130 μmol/Kg	Phenol-Explorer
	50 μmol/Kg	[74]
Onion, white	65 μmol/Kg	Phenol-Explorer
	1.2 μmol/Kg	[74]
Pear	3 μmol/Kg	[56]
Raspberry	270 μmol/Kg	[56]
Shallot	65 μmol/Kg	Phenol-Explorer
Sorghum	165 μmol/Kg	Phenol-Explorer
Strawberry	112 μmol/Kg	[56]
Star anise	2,090 μmol/Kg	Phenol-Explorer
Wine, red	0.3-0.8 μmol/L	[79]
Wine, white	0.1-0.5 μmol/L	[79]

*Values given are for the free phenolic as well as the phenolic derived from either esters or glycosides. All published values converted to μmoles per unit volume or per Kg fresh fruit (based upon an 85% water content) or per Kg grain.

^†^References for Phenol-Explorer are: [80-82].

Protocatechuic acid has anti-inflammatory activity [71] and activates Nrf2 [69] through Jun kinase (JNK) modification of the Nrf2 signalling system [83]. In this in vitro study, 25 μM protcatechuic acid was used. However, a lower concentration (3 μM) demonstrated an enhancement in the antioxidant defense systems [84]. In another in vitro assay the concentration of protocatechuic acid required to double the quinone oxireductase activity in murine hepatoma cells was 4.3 μM [69]. These studies suggest that diet may result in plasma protcatechuic acid levels sufficient to enhance the antioxidant defense systems. Protocatechuic acid also has antihyperglycemic effects in streptozotocin-induced diabetic rats [85], possibly through activation of the Nrf2 system. For a detailed discussion on the potential role of protocatechuic acid in preventing disease or treating disease see [71].

#### ***3-Monohydroxybenzoic Acid (3-MHBA) and 3,5-Dihydroxybenzoic Acid (α-Resorcylic Acid)***

The final compounds to be considered are 3-MHBA (also known as *m*-hydroxybenzoic acid) and 3,5-DHBA since there is an intriguing article demonstrating that they are agonists of the hydroxycarboxylic acid receptors HCA_1_ and HCA_2_[86]. The HCA receptor family is G-protein coupled (G_i_) and comprised of three members: HCA_1_, HCA_2_ and HCA_3_[86] predominantly expressed on adipocytes. Activation of HCA receptors inhibits lipolysis. They were formerly classified within the nicotinic acid receptor family. The natural ligand for HCA_1_ appears to be lactic acid (EC_50_ = 1.3-4.8 mM) whose normal plasma concentrations (in low mM range) can activate HCA1 to decrease lipolysis. The natural ligand for HCA_2_ is 3-hydroxybutyric acid (EC_50_ = 0.7-0.8 mM) whose plasma levels can reach 6–8 mM during fasting. Nicotinic acid also acts as a ligand for HCA_2_ and has been used pharmacologically to treat dyslipidemia [87], although it has an associated flushing problem. The natural ligand for HCA_3_ is 3-hydroxyoctanoic acid (EC_50_ = 4–8 μM) whose levels rise during starvation and diabetic ketosis [86]. These receptors are, thus, intimately involved in the feedback mechanisms regulating lipolysis.

3-MHBA is an agonist for both HCA_1_ (EC_50_ of 186 μM) and HCA_2_ (EC_50_ of 158 μM while 3,5-DHBA is a specific agonist for HCA_2_ (EC_50_ of 172 μM). Activating HCA_1_ and HCA_2_ inhibits lipolysis in adipocytes [86]. These data suggest that altering diet to include 3-MHBA and/or 3,5-DHBA may help control dyslipedemia. However, little information is available regarding the presence of these hydroxybenzoic acid compounds in the plants we eat. A little more is known about 3-MHBA (see Table 3) than 3,5-DHBA.

**Table 3:** Dietary sources of 3-monohydroxybenzoic acid

**Source**	**Content**	**References**
Avocado	62 μmol/Kg	[73]
Beer	6 μmol/L	[57]
Blueberry, *V. arctostaphylos*	1.5 μmol/Kg	[60]
Cranberries, fruit	27 μmol/Kg	Phenol-Explorer
	66 μmol/L	[42]
Medlar, ripe	0.7 μmol/Kg	[77]

#### ***Important sources of hydroxybenzoic acids are microbial metabolites of more complex phenolics***

Zhang and colleagues used a commercial cranberry drink to determine the proportion of dietary phenolics transferred to the blood [41]. The cranberry drink was comprised of 27% juice and contained 2.41 μg 2,3-DHBA/mL [41] with a total of 1800 mL consumed by each test subject (i.e., a total of 438 μg or 2.84 μmoles 2,3-DHBA). After 45 minutes blood was taken and plasma level of phenolics were determined. At this time plasma levels of 2,3-DHBA was 2.06 μg/mL. Even if the 2,3-DHBA was solely restricted to plasma and not cells or other body fluids, this is a greater amount of 2,3-DHBA than what was consumed. One can only conclude that there is metabolism of other phenolics to 2,3-DHBA, likely by gut bacteria. Indeed, there is an abundance of evidence that gut bacteria metabolize more complex phenolics such as flavonoids into simpler phenolics [88].

Another example is 3,4-DHBA (protocatechuic acid) which can be an oxidation product of the flavonoid quercetin [89] as well as a microbial metabolite of catechin [90] and anthocyanins and procyanidins [91]. Humans fed 60 g/day of a black raspberry freeze-dried powder, rich in anthocyanins, achieved a mean protocatechuic acid plasma level of 25 nM [92]. In another study where human participants ate two portions of a variety of small berries daily achieved mean protcatechuic acid plasma levels of 130 nM [93]. A third study where elderberry extract containing a total of 500 mg anthocyanins was consumed daily, protocatechuic sulfate plasma levels reached 360 nM three hr after intake [94]. A fourth study had humans consume 1 liter of blood orange juice rich in cyanidin glucosides - here a plasma level of 0.5 μM protocatechuic acid was observed 2 hr following ingestion [91]. Whether intake of protocatechuic acid via the diet will result in sufficient plasma concentrations to have a pharmacological effect is not yet demonstrated, but as noted below perhaps it can have a tipping effect.

### Phytochemicals as tipping point compounds rather than pharmaceuticals

Disease is a deviation from homeostasis. In a ‘normal’ diet it is rare that one can consume enough of a given food to achieve a plasma concentration of a specific compound of interest to have a pharmacologically significant effect. However, one must keep in mind that we are constantly consuming foods that have more than one of these compounds that can affect, for example, the Nrf2 system. An increase in the consumption of anyone of these may be the tipping point to activate the Nrf2 system sufficiently to result in cells with a more normalized redox status. For example, for Nrf2 to translocate from the cytoplasm to the nucleus requires oxidation of thiols on Kelch-like ECH-associated protein-1 (Keap1), the protein that anchors Nrf2 to the cytoskeleton, but the phosphorylation status of particular amino acid residues on Nrf2 also determines the efficacy of nuclear translocation [95]. The action of sulforaphane is oxidation of Keap1 thiols [96], whereas the action of protocatechuic acid is on the phosphorylation status of Nrf2 [83]. Thus, protocatechuic acid will enhance the efficacy of low levels of an inducer such as sulforaphane. It may well be possible that on a background of a diet containing low levels of sulforaphane glucosinolate (that in itself has no significant effect on the activation of the Nrf2 system) that consuming low levels of protocatechuic acid may be the tipping point towards activation (i.e., nuclear translocation) of the Nrf2 system resulting in a more normal redox state for cells.

Similarly, the EC_50_s of 3-MHBA for HCA_1_ and 3,5-DHBA for HCA_2_ suggest that diet cannot influence the activation state of these hydroxycarboxylic acid receptors. Although the EC_50_ is an important measure of activity since it is a measure of the concentration where 50% of the receptors are activated, it is not a measure of the kinetics of the binding. Importantly it does not measure the time a compound occupies and activates the receptor. If, for example, the 3,5-DHBA-HCA_2_ dissociation time is significantly longer than the lactate-HCA_2_ dissociation time, then this effectively lowers that lactate concentration necessary to activate the HCA_2_ signalling pathway. It is very possible that concentrations of the hydroxycarboxylic acid an order or two below the EC50 will allow lower concentrations of the natural ligand to result in physiologically significant increases in receptor activation states to result in decreases in lipolysis to significantly affect blood lipid levels. In other words dietary intake of 3-MHBA and 3,5-DHBA that result in low μmolar plasma concentrations may tip the scale towards more normal lipid profiles.

## Concluding remarks

We are now at the knowledge tipping point where rather than having vague guides on eating more fruits and vegetables to improve health we can design diets to include specific phytochemicals that influence cellular signaling and gene expression. For example, diets containing specific Nrf2 activators that act on Keap1 thiols as well as activators that act on the phosphorylation states of Nrf2 allowing more efficient Nrf2 translocation to the nucleus - the end result is a more normal redox status of cells with consequences that include decreased probabilities of developing hypertension and developing atherosclerotic lesions. We can design diets that increase the content of 3-MHBA and/or 3,5-DHBA that results in tipping to a more normal blood lipid profile, again decreasing the probability of developing atherosclerotic lesions. We are at the beginning of understanding how phytochemicals may influence signaling pathways that influence cardiovascular health. We trust we have intrigued the readers sufficiently to do further research on the distribution, microbial metabolism and uptake of hydroxybenzoic acids as well as on their potential therapeutic actions.

## Abbreviations

COX: Cyclooxygenase; CYP: Cytochrome P450; DHBA: Dihydroxybenzoic acid; 2,3-DHBA: 2,3-Dihydroxybenzoic acid; 2,4-DBHA: 2,4-Dihydroxybenzoic acid; 2,5-DHBA: 2,5-Dihydroxybenzoic acid; 2,6-DHBA: 2,6-Dihydroxybenzoic acid; 3,4-DHBA: 3,4-Dihydroxybenzoic acid; 3,5-DHBA: 3,5-Dihydroxybenzoic acid; EC_50_: Half-maximal effective concentration; G_i_: Guanosine-nucleotide-binding protein alpha inhibitory; G-protein: Guanosine nucleotide-binding protein; HCA: Hydroxycarboxylic acid receptor; 3-MHBA: 3-Monohydroxybenzoic acid; mM: Millimolar; μM: Micromolar; nM: Nanomolar; NFκB: Nuclear factor kappa B; Nrf2: Nuclear factor (erythroid-derived-2)-like-2.

## Competing interests

The authors declare that they have no competing interests.

## Authors’ contributions

The concept for this paper was developed by PG. HJA, AMZA and BMHA did extensive literature research and wrote the first draft of the manuscript. BHJJ revised the manuscript, in particular adding background on Nrf2 activators and HCA activators. All authors read and approved the final manuscript.

## Authors’ information

Dr P. Ganguly (MBBS, MD, FACA) has spent many years in catecholamine research in health and disease. He worked earlier with metabolites of catecholamines and found that oxidation products such as adrenochrome may be detrimental to cardiac function. B.H.J. Juurlink (PhD) has spent many years examining how cellular redox influences inflammation and how phytochemicals can promote a more normal redox environment through Nrf2 activation thereby decreasing aging-associated problems such as hypertension and generalized inflammation. Ms Azouz, Ms Aldalati and Ms AlTinawi are second year medical students with an interest in how dietary phytochemicals may influence health.
